# From Design to Bedside: Brain-Targeted Engineered Nanoparticles and Predictive Modeling for Neurodegenerative Diseases Therapeutics

**DOI:** 10.1007/s11095-026-04201-5

**Published:** 2026-10-01

**Authors:** Nguyen Nguyen, Muzhaozi Yuan, Ya Wang

**Affiliations:** 1 J. Mike Walker ‘66 Department of Mechanical Engineering, Texas A&M University, College Station, TX 77840 United States; 2 Department of Biomedical Engineering, Texas A&M University, College Station, TX 77843 United States; 3 Department of Electrical and Computer Engineering, Texas A&M University, College Station, TX 77843 United States

**Keywords:** brain disorders, brain targeting, CNS nanomedicine, nanoparticles, neurodegenerative diseases, neurological diseases, predictive modeling, regulation

## Abstract

Nanoparticle (NP)-based drug delivery systems offer promising solutions for neurodegenerative diseases such as Alzheimer’s, Parkinson’s, and Huntington’s, which have no cure due to the brain’s structural complexity and the restrictive nature of the blood-brain barrier (BBB). Conventional therapeutics, despite strong *in vitro* efficacy, often fail clinically due to poor brain penetration. Recent advances in NP engineering, including optimization of size (10–100 nm), surface charge (-10 to -20 mV), and targeted ligand decoration, have improved BBB crossing and reduced premature clearance. In parallel, predictive in silico approaches, such as physiologically based pharmacokinetic (PBPK) modeling, computational fluid dynamics, and machine learning models, enable quantitative prediction of NP biodistribution, residence times, and region-specific uptake. By simulating key determinants, including blood flow dynamics, vasculature heterogeneity, permeability, and retention, these computational tools accelerate lead selection, reduce experimental burden, and inform design before *in vivo* testing. This review integrates recent progress in NP design with advanced computational modeling, highlighting how their convergence provides a rational framework for developing next-generation brain-targeted therapeutics. We further discuss regulatory and translational challenges, including safety, biocompatibility, and the need for humanized preclinical benchmarks. Together, these innovations offer a roadmap for advancing NP-based strategies toward clinical impact in neurodegenerative disease.

## Introduction

The way we tackle disease in human society has been dictated by the lessons which we have learned throughout medical history. The first appearance of medicinal drugs in particular presented themselves in forms of herbs, plants, roots, vines, and fungi which served as the primary source of pharmaceuticals used to relieve human pain in ancient civilizations such as Egypt, China, and India [1]. Herbal remedies were primarily obtained through practice and not through rigorously tested clinical trials. Thus, the efficacy and safety for treating specific conditions was not ensured. Synthetic drugs were introduced later in the 1800 s, along with the development of other related fields [2]. This foundation developed new medications and enhanced the effectiveness of existing drugs in a wide range of medical conditions and therapeutic applications, thereby establishing a pillar for modern medicine. Advancements in therapy have made it possible to treat a wide range of internal organs (kidneys, liver, heart, etc.). Yet, the brain, due to its immune-privileged status, remains a challenging frontier where common drugs demonstrate minimal effect. Neurodegenerative diseases are a tenacious group of disorders in the brain that cause the progressive loss of structure and function of nerve cells in the central nervous system (CNS). Since regular chemical substances cannot bypass the blood-brain barrier (BBB) due to its protective effect, scientists must turn to alternative approaches to treat neurodegenerative diseases.

Inspired by Paul Ehrlich's postulation of a “magic bullet”, the concept of using particles as drug carriers was first introduced in the 1960 s, following the emergence of nanotechnology [3]. Nanoparticles (NPs) demonstrate the potential to overcome the BBB and target specific cells or tissues in the brain, making them attractive candidates for drug delivery. Since then, NPs have been actively researched and engineered as carriers to deliver drug more effectively. There has been a plethora of studies on synthesizing new NP-based carriers and targeting complex neurological environments to treat neurodegenerative diseases such as Alzheimer's disease (AD), Parkinson's disease (PD), multiple sclerosis, etc. Subsequently, scientists have been tailoring the composition, morphology, dimensions, and surface of NPs to optimize the biodistribution of encapsulated drugs. Despite such efforts in drug development, therapies for neurodegenerative diseases continue to struggle with high failure rates in clinical trials.

This review provides a comprehensive analysis of the major breakthroughs and translational strategies in brain-specific nanomedicine developed over the past decade (2016–2026) for treating neurodegenerative diseases. While traditional literature focuses extensively on isolated pathological mechanisms, this work shifts the paradigm toward engineering innovations and predictive transport modeling essential for clinical translation. We evaluate cutting-edge material engineering strategies alongside physical interventions designed to breach restrictive biological barriers. Crucially, we highlight the pivotal role of advanced administration routes and state-of-the-art predictive modeling pipelines, spanning Pharmacokinetics & pharmacodynamics, Physiologically-based pharmacokinetics, Computational Fluid Dynamics, and Artificial Intelligence/Machine Learning. By integrating human-centric physiological systems with these multiscale computational tools, this review offers a unique blueprint for mapping real-world human biodistribution, navigating regulatory frameworks under the FDA Modernization Act 2.0, and accelerating the clinical entry of next-generation central nervous system therapeutics (Table 1).

**Table 1:** A comparative analysis of key themes and limitations in current review papers on NP-based drug delivery for neurological diseases highlights the unique contributions

Primary focus	Key contributions	Limitations	This review's unique contributions
Recent Progress of Nanomedicine in the Treatment of AD (2023) [4]	Nanomedicine efficacy in AD, BBB penetration strategies, therapeutic nanoparticle design	Lack emphasis on personalized medicine and regulatory pathways for clinical translations	Integrate predictive modeling tools and emphasize the importance of comprehensive regulatory considerations
Nanomedicine for Neurodegenerative Disorders (2021) [5]	Neurodegenerative disease pathology and nanoparticle applications	Broad coverage but lack focus on engineering innovations and their mechanistic insights	Discuss NP delivery challenges (i.e. BBB crossing) while specifying NP engineering innovations to address these challenges
Current Overview on the Use of Nanosized Drug Delivery Systems for Neurodegenerative Diseases (2024) [6]	Drug delivery challenges and non-invasive administration approaches	Limited discussion on the engineering approaches and quantitative metrics to assess delivery efficiency	Highlight the role of predictive modeling tools in optimizing drug delivery systems, a focus largely absents in other reviews
The Blood-Brain Barrier: Structure, Regulation and Drug Delivery (2023) [7]	BBB structure, regulation for permeability, and transport mechanisms	Limited discussion of advanced simulation platforms and predictive modeling for NP transport	Provide an integrated look at how predictive modeling can be utilized to anticipate NP biodistribution and efficacy
Nanoparticles for Targeted Brain Drug Delivery: What Do We Know? (2021) [8]	Therapeutic NP design, active-targeted delivery strategies, and potential in treating neurological diseases	While comprehensive, primarily focuses on structural aspects without practical integration with NP technologies	Consolidate the existing knowledge of BBB mechanisms with practical implications for NP design and therapeutic targeting. Investigate how predictive modeling can be utilized to anticipate NP biodistribution and efficacy

In summary, this review provides a forward-looking perspective that bridges the gap between experimental research and real-world applications by highlighting the clinical challenges and regulatory frameworks necessary for successful translation from preclinical to clinical stages. In this context, our review offers a comprehensive overview of advancements in nanomedicine, unique insights into the predictive modeling tools and engineering innovations that are poised to shape the future of brain-targeted therapies for neurodegenerative diseases. This makes it an indispensable resource for researchers seeking to optimize NP-based drug delivery systems and clinicians aiming to bring these cutting-edge treatments to patients in the future.

## Biological Barriers and Region-Specific Therapeutic Challenges in Neurodegenerative Diseases

### Region-Specific Neurodegeneration and Therapeutic Limitations

Neurodegenerative diseases are progressive disorders characterized by the gradual loss of neurons, leading to cognitive decline, motor dysfunction, and behavioral alterations. Despite differences in etiology, AD, PD, HD share several pathological features, including protein aggregation, neuroinflammation, oxidative stress, and progressive synaptic degeneration. Importantly, the regional specificity of these pathologies presents a major obstacle for therapeutic intervention, as effective treatment requires sufficient drug accumulation within localized and often difficult-to-access areas of the central nervous system. Understanding the underlying pathologies is crucial for advancing nanomedicine-based interventions.

**Fig. 1:**
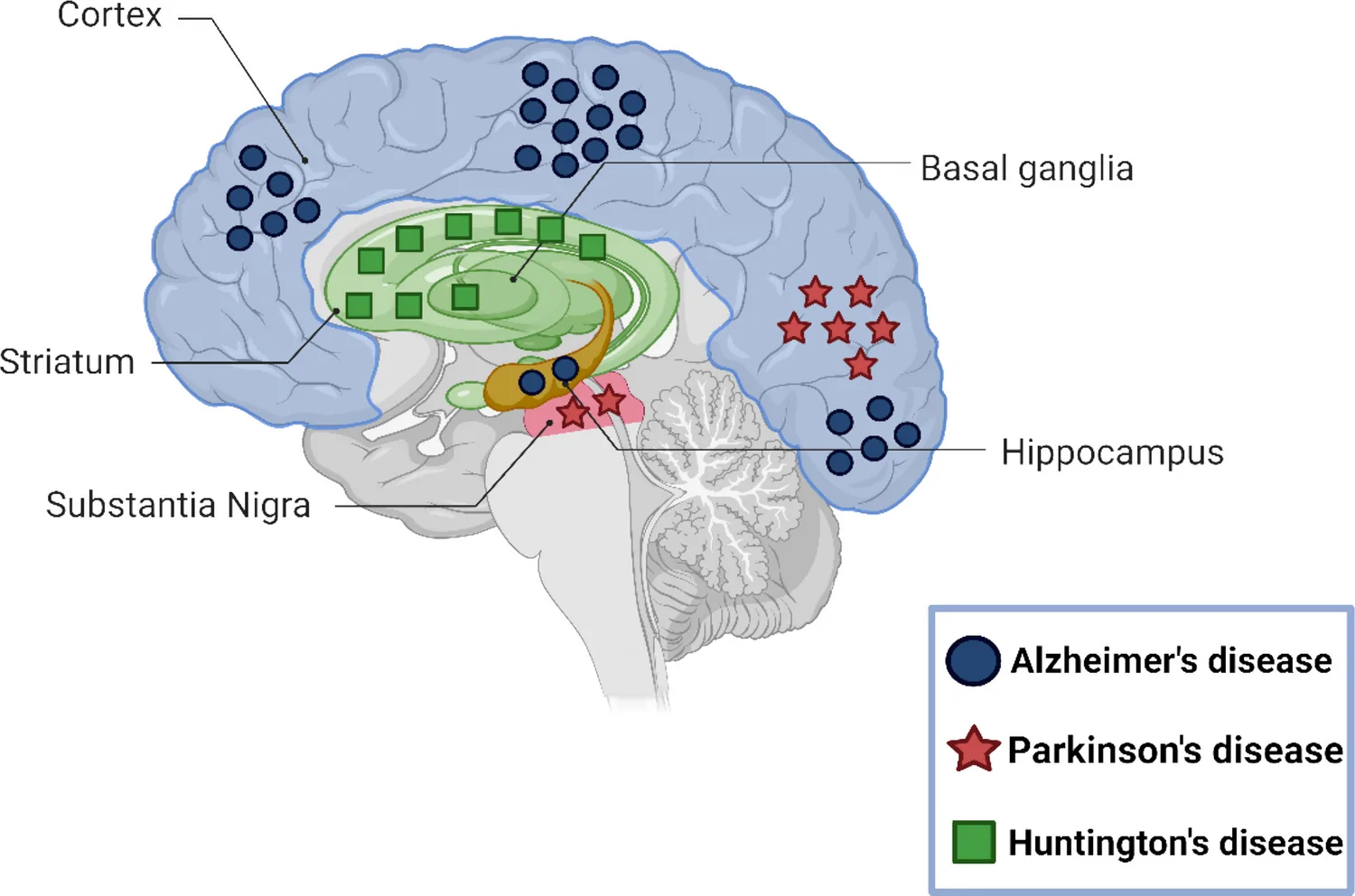
Affected regions of different neurodegenerative diseases.

AD is primarily associated with the accumulation of amyloid-β (Aβ) plaques and tau neurofibrillary tangles within the hippocampus and cortical regions (Fig. 1), resulting in synaptic dysfunction and progressive cognitive decline [9, 10]. Current therapeutic strategies, including cholinesterase inhibitors [11]^11^,^13^, NMDA receptor antagonists [14], and anti-amyloid monoclonal antibodies, provide modest symptomatic or disease-modifying benefit but remain limited by poor BBB penetration and systemic adverse effects.

Similarly, PD is characterized by degeneration of dopaminergic neurons within the Substantia Nigra Pars Compacta (SNpc), a subregion in the midbrain and striatum (Fig. 1), that is accompanied by α-synuclein aggregation and chronic neuroinflammation [15, 16]. Although dopamine replacement therapies such as levodopa remain the clinical standard for symptom management [17], long-term administration is associated with motor complications and does not prevent ongoing neurodegeneration [18].

In HD, expanded cytosine-adenine-guanine (CAG) repeats within the huntingtin gene produce mutant huntingtin protein aggregates that selectively damage medium spiny neurons in the striatum (Fig. 1), leading to progressive motor and psychiatric dysfunction. Existing therapies largely focus on symptomatic management and fail to address the underlying genetic pathology [19, 20] (Table 2).

**Table 2:** FDA-approved neurodegenerative drugs

Disease targeted brain region	Therapeutic category	Drug name	Effective dosage	Therapeutic effect
AD: Hippocampus, and Cortex	Disease-Modifying	Aducanumab (Aduhelm®)	10 mg/kg IV monthly [21]	Targets Aβ plaques; risk of ARIA
		Lecanemab (Leqembi®)	10 mg/kg IV biweekly [22]	Reduces amyloid levels; modest clinical benefit
	Symptomatic Treatment	Donepezil (Aricept®)	10 mg daily [11]	Cholinesterase inhibitor; improves cognition
		Rivastigmine (Exelon®)	12 mg daily (oral) or 15 cm^2^ patch[12]	Higher GI side effects; cognitive function
		Galantamine (Razadyne®)	24–32 mg daily [13]	Cholinesterase inhibitor; cognitive improvement
		Memantine (Namenda®)	20 mg daily (with Donepezil 10 mg) [14]	NMDA receptor antagonist; combined therapy for moderate-severe AD
PD: SNpc	Symptomatic Treatment	L-DOPA (Sinemet®)	25/100 mg three times daily [17]	Standard therapy for motor symptoms; dose adjusted based on response
		Pramipexole (Mirapex®)	0.125 mg three times daily [23]	Dopamine agonist; titrated to effect; side effects include impulse control disorders
		Ropinirole (Requip®)	0.25 mg three times daily[24]	Dopamine agonist; titrated weekly; may cause hallucinations
		Selegiline (Eldepryl®)	5 mg twice daily [25]	MAO-B inhibitor; taken with breakfast and lunch
		Rasagiline (Azilect®)	1 mg once daily[26]	MAO-B inhibitor; helps prolong dopamine action
HD: Striatum	Symptomatic Treatment	Tetrabenazine (Xenazine®)	Start at 12.5 mg daily, up to 100 mg/day[19]	Manages chorea; risk of depression and parkinsonism
		Deutetrabenazine (Austedo®)	Start at 6 mg twice daily [27]	Similar to Tetrabenazine; lower risk of side effects
		Sertraline (Zoloft®)	Start at 50 mg daily [28]	SSRI for psychiatric symptoms; dose adjusted as needed

### Physiological and Biological Constraints Governing NP Brain Delivery

Although nanotechnology offers a groundbreaking approach to targeting the brain, achieving consistent and reliable delivery remains a significant obstacle. After nearly five decades of research, the overall efficiency of NP-based systems in delivering therapeutic agents to the brain remains disappointingly low. Only a fraction (~ 2% of IV/oral) of the administered dose successfully reaches the target site, as NPs must navigate a series of dynamic physiological and biological barriers before accumulating within diseased brain regions. The transport behavior of NPs is therefore governed not only by their intrinsic physicochemical properties, but also by continuous interactions with biological environments throughout circulation and tissue penetration.

**Fig. 2:**
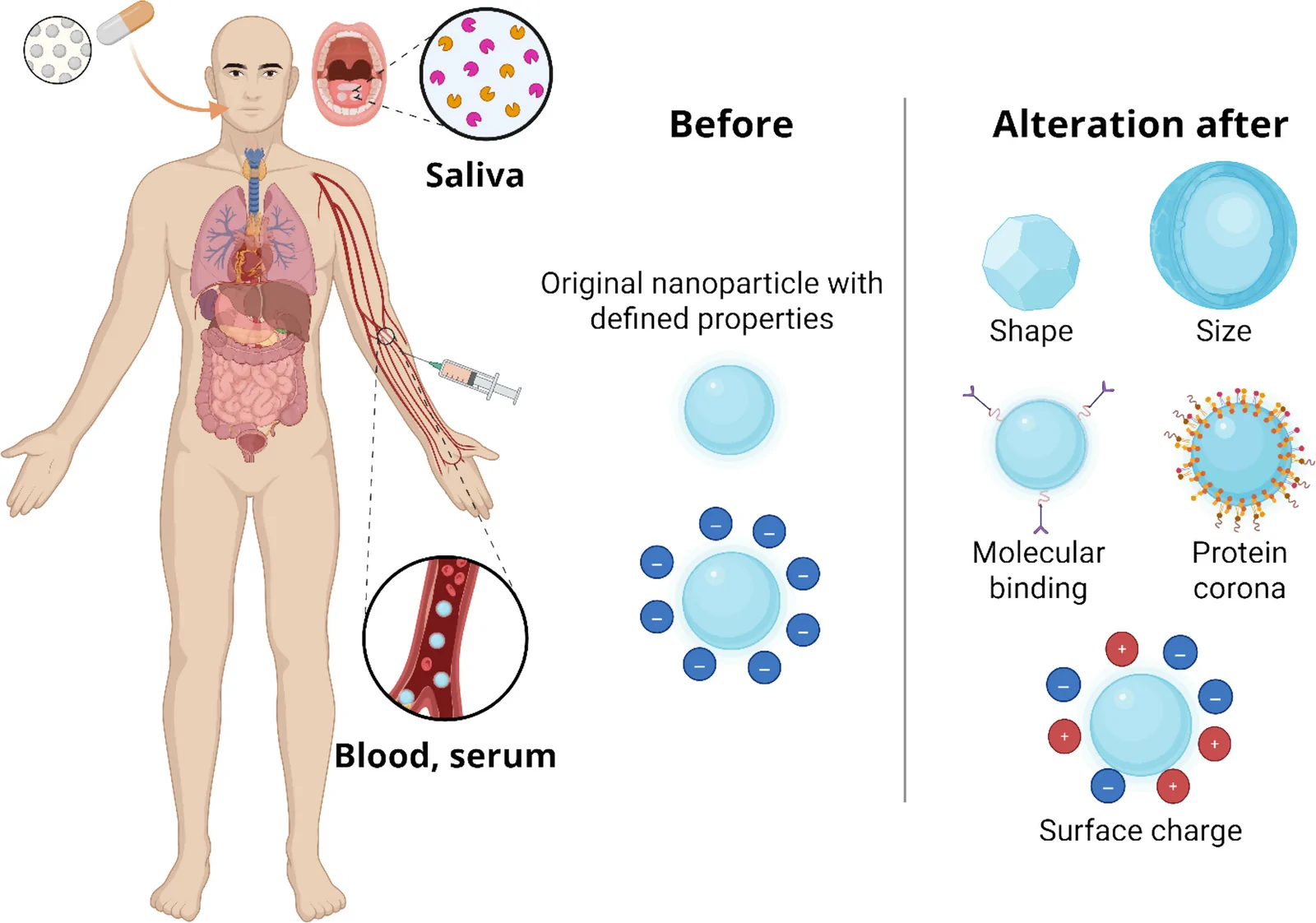
Surface alteration of NP delivery systems after transport to multiple compartments.

Immediately after administration, NPs encounter complex biological fluids that rapidly alter their surface characteristics through adsorption of proteins and biomolecules [29, 30], forming a “protein corona” as shown in Fig. 2. This process changes NP size, surface charge, colloidal stability, and biological identity, thereby influencing endothelial uptake, immune recognition, biodistribution, and systemic clearance [31]. Protein corona formation is highly dynamic and depends on both NP composition and the surrounding physiological environment. For example, 15 min after administration, the cerebrospinal fluid-derived protein coronation in polymer NPs preferentially promotes their internalization into brain cells. [32] Similarly, gold NP interaction with different proteins has also shown changes in NP hydrodynamic size and charge depending on various shapes [33]. For another example, the uptake of gold NP into endothelial cells (ECs) has been increased by platelet factor 4 proteins binding to the gold NP surface, forming a protein corona [34]. In some cases, this effect can trigger recognition by the immune system, leading to rapid clearance by phagocytic cells such as macrophages and monocytes [35].

As NPs transit through circulation and intracellular compartments, continuous exchange of adsorbed biomolecules due to low pH and high enzyme concentrations may further modify transport behavior and therapeutic performance [36]. In parallel, intracellular trafficking through acidic endosomal and lysosomal compartments can induce NP degradation, aggregation, or premature drug release [37], particularly in polymeric biodegradable and inorganic nanocarriers such as poly (D, L-lactide-co-glycolide) (PLGA) and gold or silica NPs [38, 39].

Beyond these nanoscale interactions, vascular and disease-specific physiological factors further complicate delivery efficiency. Cerebral blood flow regulates NP margination, endothelial adhesion, and vascular residence time, directly affecting the probability of BBB interaction and cellular internalization. High blood flow velocity can hinder binding to the BBB, reducing effectiveness, while low velocity may increase residence time and uptake. Microfluidic studies have confirmed that reduced flow enhances NP internalization into endothelial cells, highlighting the importance of circulation dynamics within the brain vasculature in delivery efficiency [40]. Simultaneously, neurodegenerative diseases exhibit substantial spatial and temporal heterogeneity, where pathological regions evolve progressively during disease progression [41]. The clinical symptoms often mirror the anatomical trajectory of degeneration, indicating a constantly shifting pathological landscape. As the disease progresses, the ratio of diseased to healthy cells evolves, creating a moving target for NP-based therapies. As a result, NPs must selectively localize within relatively small and dynamically changing populations of diseased neurons while minimizing off-target accumulation in healthy tissue. This challenge is particularly important in disorders such as AD, PD, and HD, where pathological burden is unevenly distributed across distinct brain regions and disease stages.

**Fig. 3:**
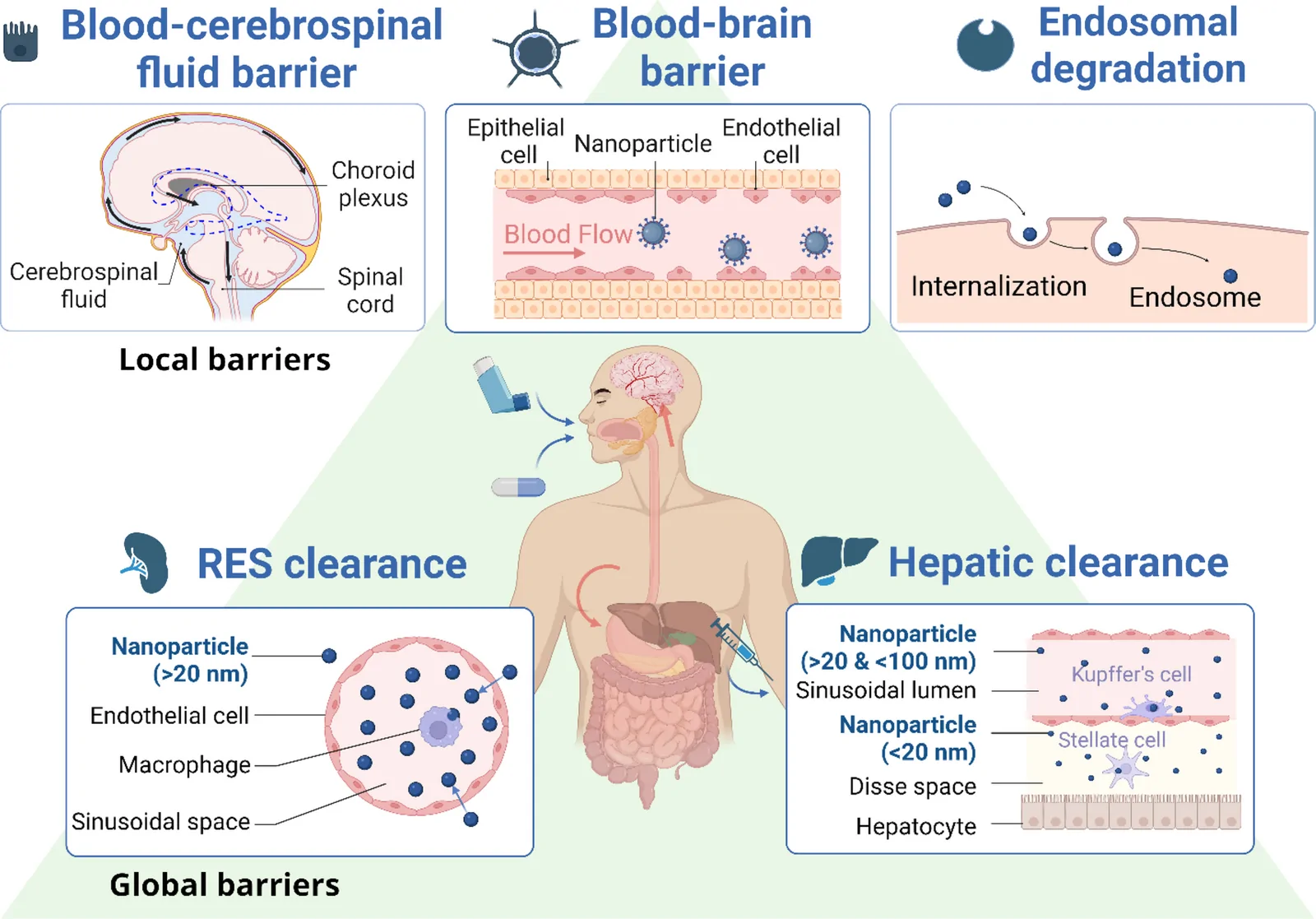
Different levels of biological barriers for nanoparticle drug delivery systems.

In addition to these transport limitations, brain delivery is further restricted by highly selective biological barriers, including the reticuloendothelial system (RES), blood–brain barrier (BBB), and blood–cerebrospinal fluid barrier (BCSFB) (Fig. 3). The RES rapidly removes circulating foreign particles through phagocytic clearance in organs such as the liver and spleen, substantially reducing systemic circulation time [42]. Meanwhile, the BBB and BCSFB tightly regulate molecular transport into the CNS through endothelial tight junctions, active efflux transporters, and limited transcytosis activity [43].

Collectively, these multiscale barriers severely limit NP accumulation within the brain and remain one of the primary reasons for the poor clinical translation of CNS-targeted nanomedicine. Successful brain-targeted NP systems require careful integration of Adaptive or multi-targeted delivery strategies from material engineering, surface functionalization, and predictive transport modeling to optimize circulation stability, barrier penetration, and region-specific therapeutic delivery.

### BBB Dysfunction in Neurodegenerative Diseases

**Fig. 4:**
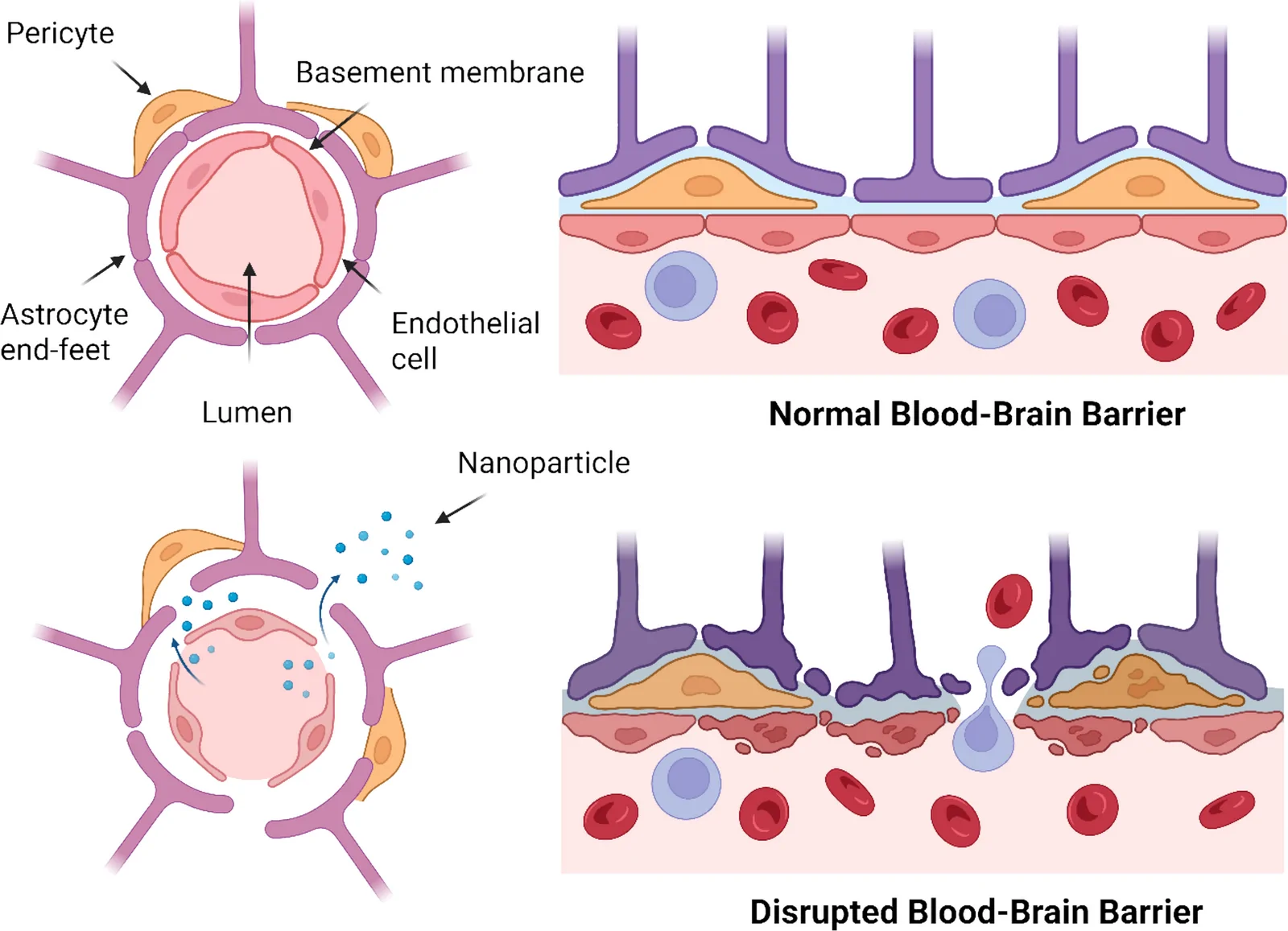
Comparison of the BBB in Normal and Pathological Conditions. Top: In the healthy brain, the BBB comprises tightly connected endothelial cells, pericytes, and astrocytic end feet, maintaining selective permeability and protecting the brain microenvironment. Bottom: Under pathological conditions, the BBB becomes disrupted, leading to endothelial dysfunction, pericyte detachment, astrocyte reactivity, and increased permeability.

Covering 99% of the cerebral capillaries, the BBB only allows small, lipid-soluble molecules (such as oxygen and certain hormones) to pass through [44] while actively excluding macromolecules and neurotoxins via efflux transporter [45] and enzymatic degradation, such as monoamine oxidase [46] (Fig. 4). Although this protective function is essential for maintaining neuronal stability, it also represents one of the greatest obstacles to effective brain-targeted drug delivery, as the vast majority of therapeutics and nanocarriers exhibit limited BBB penetration under physiological conditions. As a result, many nanoparticle-based therapeutic systems that demonstrate strong *in vitro* efficacy fail to achieve sufficient *in vivo* brain accumulation due to inadequate transport across the BBB and vascular components.

In neurodegenerative diseases, BBB integrity becomes progressively altered by chronic inflammation, oxidative stress, endothelial dysfunction, and pathological protein aggregation. Disorders such as AD, PD, [47], multiple sclerosis [48], hypoxic and ischemic insult [49], and epilepsy [50], exhibit variable BBB disruption, ranging from minor and temporary alterations in BBB permeability due to ECs deterioration [51, 52], to chronic barrier breakdown that dysregulates the systems and enzymes [51, 53].

Early detection of BBB disruption in clinical settings requires imaging techniques to assess vascular leakage or capture physiological alterations. Techniques such as dynamic contrast-enhanced magnetic resonance imaging (DCE-MRI) provide quantitative assessment into vascular integrity and regional BBB disruption. Permeability can increase approximately 190% in the hippocampus for AD [54] and 20–50% in the substantia nigra in PD [55], highlighting the distinct regional vulnerabilities of the BBB in these conditions. Meanwhile, emerging *in vitro* and computational BBB models also enable mechanistic investigation of nanoparticle transport and biodistribution. Together, these approaches become increasingly important for the rational design of nanomedicine systems to enable navigating them inside the complex and evolving BBB microenvironment.

## NP Drug Delivery System & Engineering Approaches to Improve Targeting

Building upon the challenges of brain-specific drug delivery, recent advances in NP design offer new possibilities for improving BBB penetration and achieving precise targeting of diseased brain regions [56]. This section examines cutting-edge strategies in NP delivery systems, highlighting their potential to overcome existing barriers and enhance therapeutic outcomes for neurodegenerative conditions.

### Improving the BBB Permeability

The BBB’s restrictive nature demands innovative strategies. One prominent approach is ligand functionalization to engage receptor-mediated transcytosis pathways. Transferrin (TfR), lactoferrin (Lf), Low-Density Lipoprotein receptor-related protein 1 (LRP-1), and insulin receptors, as well as peptides and antibodies, are among the most studied. For instance, TfR- conjugated with PLGA NPs [57], and Lf-modified NPs [58], showed significantly enhanced uptake in mice. NPs functionalized with peptides or antibodies targeting LRP-1 [59] and Insulin [60, 61] have also successfully crossed the BBB and reduced inflammation in the brains of AD mice. However, challenges remain, including short serum half-lives, competition with endogenous ligands in the bloodstream for receptor-binding sites, and limited receptor specificity. Researchers are exploring different antibodies against these receptors as alternative ligands to circumvent competition and enhance BBB crossing efficacy. Ideally, target receptors for BBB crossing would be highly expressed on brain ECs while being minimally present on other vascular ECs, reducing off-target effects and potential safety concerns. However, few receptors currently meet this ideal criterion. The choice of ligand will depend on the specific target and the type of disorder being targeted. Therefore, the effectiveness and specificity of ligand-modified NPs need to be tested rigorously in laboratory and clinical studies before they can be used in medical treatment.

Beyond biochemical targeting, physicochemical tuning is equally essential. NPs in the 10–100 nm size exhibit optimal brain uptake, as their small size facilitates passage through the EC tight junction of the BBB [62]. Surface charges around −10 to −20 mV have demonstrated superior BBB passage by minimizing electrostatic repulsion between negatively charged NPs and EC membranes, as well as aggregation [63].

**Fig. 5:**
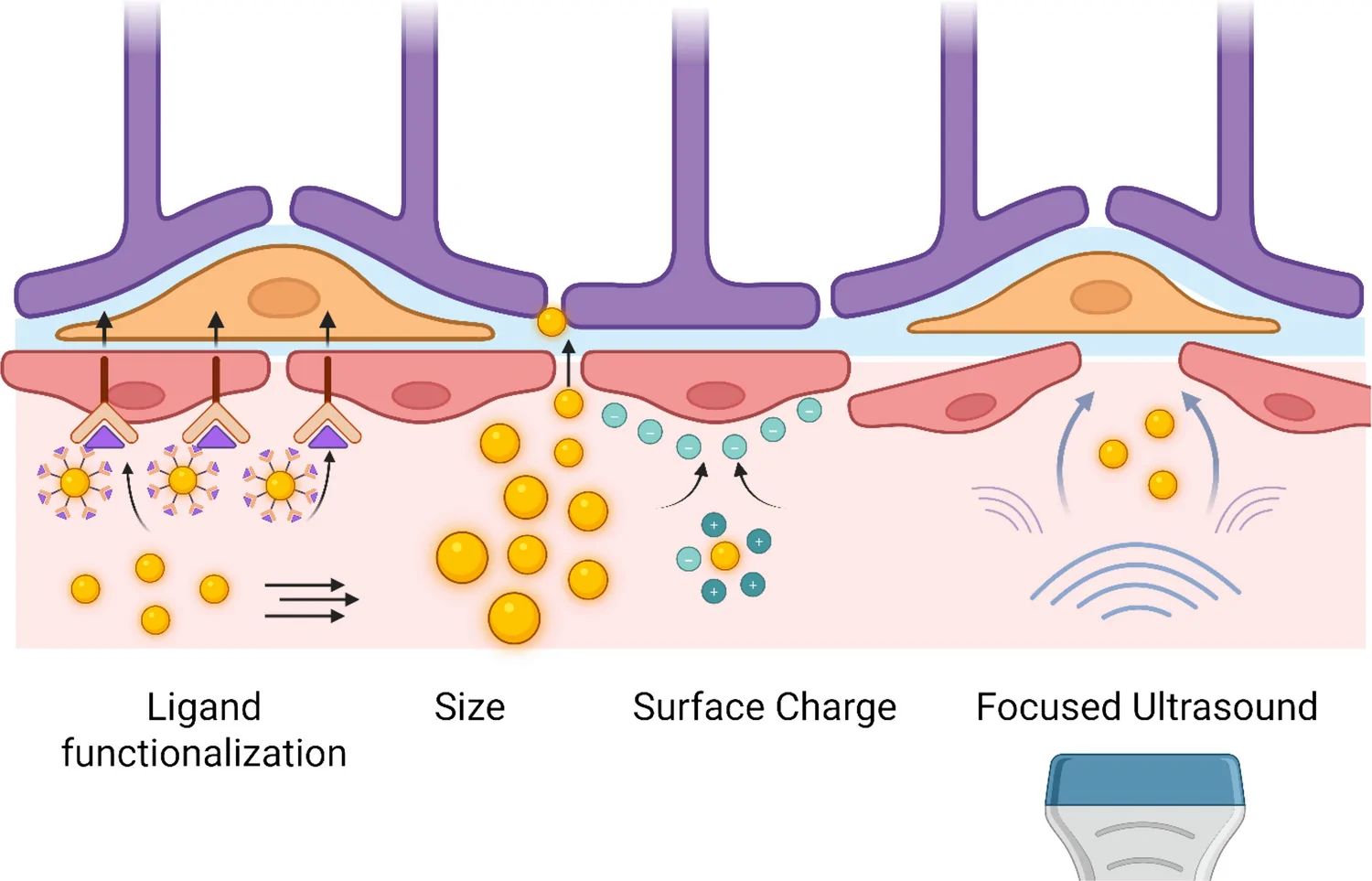
Approaches for overcoming the BBB in nanoparticle drug delivery. This figure illustrates key strategies to enhance the transport of therapeutics across the BBB, including (i) Ligand-Mediated Targeting, where NPs are functionalized with specific ligands (e.g., transferrin, lactoferrin, LRP-1, insulin) to engage receptor-mediated transcytosis pathways for efficient brain uptake; (ii) Optimization of Physicochemical Properties, including the adjustment of NP size (10–100 nm) and surface charge to facilitate BBB penetration via paracellular pathways and reduced electrostatic repulsion; and (iii) Focused Ultrasound, a non-invasive method that temporarily opens the BBB by disrupting tight junctions, enabling targeted drug delivery to specific brain regions.

A more recent non-invasive strategy is focused ultrasound (FUS), which transiently disrupts the tight junctions between ECs [64], enabling localized BBB opening and improved NP penetration (Fig. 5). Although promising, FUS requires further optimization to ensure the safe and uniform distribution of therapeutic agents and minimize adverse effects, such as tissue damage and inflammation [65].

Together, these strategies—ligand functionalization, size/charge optimization, and FUS — represent the leading approaches to enhancing BBB permeability and advancing NP-mediated therapies for neurodegenerative diseases.

### Precision Targeting of Diseased Brain Regions

Precise delivery to affected brain regions is essential for maximizing efficacy and minimizing side effects. Active targeting employs ligands such as Aβ-specific antibodies to direct NPs to pathological sites in AD, while passive targeting can exploit compromised BBB integrity and vascular permeability during neuroinflammation. However, passive strategies often lack consistency and precision, which are necessary for optimal therapeutic outcomes.

**Fig. 6:**
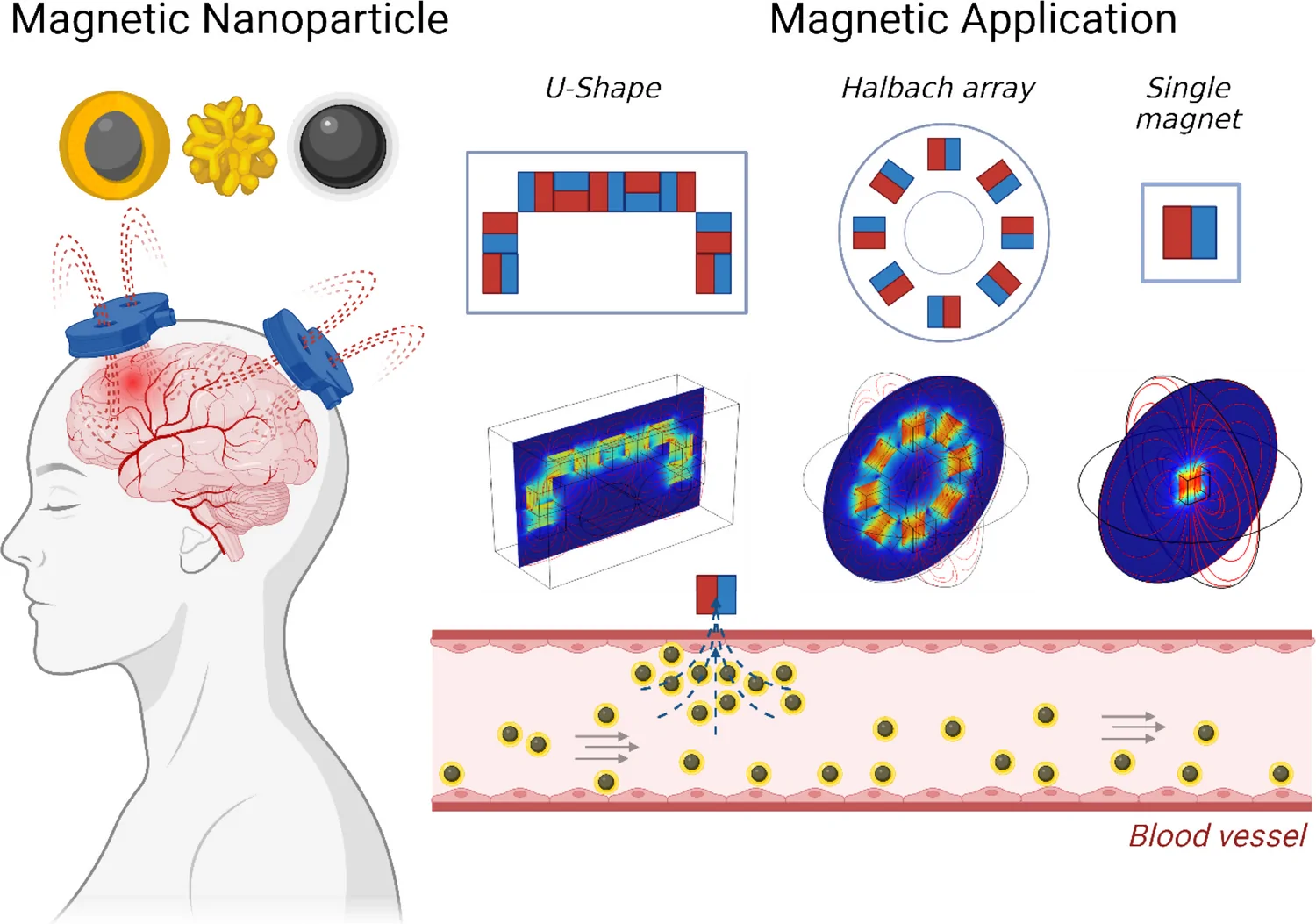
Different targeting strategies for brain region-specific delivery.

Magnetic targeting offers a promising non-invasive approach to address this issue leveraging material’s property (Fig. 6). Magnetic nanoparticles (MNPs), such as superparamagnetic iron oxide NPs (SPIONs), can be guided to specific brain areas using external magnetic fields [66], enabling spatial control of delivery. Preclinical studies in mice have demonstrated that approximately 10% of the intravenously injected MNPs reach the brain after 3000 Oe/cm exposure for 30 min [67] and their distribution across neurons, astrocytes, and microglia [68]. A more recent study in non-human primates has shown enhanced brain accumulation using a static magnetic field [69]. Surface coating, such as gold or silver, can strengthen the stability of the MNP system and improve electrical conductivity throughout the entire delivery assembly [70, 71].

Despite these advances, limitations remain. The brain is relatively low susceptible to the magnetic field. Meanwhile, concerns regarding MNP-induced toxicity, iron imbalance, oxidative stress, and altered gene expression and signaling pathways [72] must also be carefully addressed. Therefore, continued refinement of MNP design and magnetic field control will be crucial for translating this into clinical uses.

### Efficient Controlled Drug Release

The therapeutic success of NP-based therapies depends on their ability to achieve sustained and localized drug release, a crucial need in neurodegenerative diseases. Stimuli-responsive NPs can release drugs in response to local CNS cues, such as pH variations, enzymatic activity, and redox conditions [73]. For instance, NPs coated with a pH-sensitive polymer, poly(ethylene glycol)-*b*-poly[4-(2,2,6,6-tetramethylpiperidine-1-oxyl)aminomethylstyrene], and a cholinesterase enzyme-responsive supramolecular vesicle [74] have been shown to improve targeted release in AD and PD animal models, while reducing systemic toxicity.

Emerging light-responsive NPs offer even finer control. Gold or semiconductor NPs [75] can be engineered to release drugs upon near-infrared light stimulation, enabling precise spatial and temporal regulation while enhancing BBB penetration. For example, hollow gold nanospheres (HGNS)-loaded biodegradable PLGA microspheres can modulate drug release for PD medicine in response to near-infrared light exposure in preclinical studies, highlighting the potential for highly localized treatment. While promising, light-triggering NPs are still in an early stage of development, with challenges including phototoxicity and tissue safety that require further *in vivo* evaluation.

### Biocompatibility and Safety Optimization

Ensuring biocompatibility and minimizing potential toxicity is paramount for the successful clinical translation of NP-based therapies. Toxicological effects vary significantly depending on the NP composition. For instance, metal-based NPs, such as those made of gold or silver, are known to induce oxidative stress and inflammatory responses, leading to neurotoxicity [76]. To mitigate these risks, researchers have turned to biocompatible carriers such as metal-organic frameworks (MOFs) [77], PLGA [78], chitosan [79], lipids, and other organic nanocarriers [80], which are metabolized and cleared naturally to reduce the risk of chronic toxicity. MOFs, in particular, can be engineered to degrade into non-toxic components under physiological conditions, while maintaining drug delivery efficacy.

Furthermore, reducing the immunogenicity of NPs is equally critical to prevent adverse immune responses that could compromise both the safety and effectiveness of the therapy. Surface modification techniques, notably PEGylation, have been extensively employed to enhance NP stability and circulation, with Polyethylene glycol (PEG) coatings providing a hydrophilic “stealth” layer that reduces RES clearance. The clinical success of PEGylated formulations, exemplified by the FDA-approved Doxil® underscores the efficacy of this approach. Supporting this, studies by Lipka *et al*. demonstrated that > 95% of PEGylated gold NPs (10 kDa PEG) remained in circulation one hour post-IV injection. In contrast, Klibanov *et al*. reported circulation half-lives of 5 h for PEGylated liposomes *versus* < 30 min for unmodified ones [81].

Beyond synthetic coatings, biomimetic NPs represent a cutting-edge approach for biocompatibility and targeting. By mimicking the properties of endogenous cellular components or exploiting specific biological interactions, biomimetic NPs offer unique advantages for both navigating biological barriers and achieving precise targeting of diseased brain tissues. Coating NPs with cell-derived membranes (e.g., red blood cell or cancer cell membranes) transfers native surface proteins, enabling immune evasion, prolonged circulation, and enhanced BBB penetration [82, 83]. Meanwhile, small extracellular vesicles secreted by cells, called exosomes, have emerged as new natural carriers for intercellular communication and cargo delivery [84]. Exosome-mimetic NPs have demonstrated improved BBB penetration, enhanced cellular uptake, and prolonged retention within the brain thanks to their natural targeting and internalization mechanisms, offering a promising strategy for treating neurodegenerative diseases [85].

Rigorous preclinical evaluation of the biocompatibility and safety of NPs is vital before application in humans. Chronic exposure to NPs, even at low doses, could lead to neuroinflammation, neurodegeneration, or other detrimental effects on brain function. Therefore, *in vivo* animal studies of pharmacokinetics, biodistribution, and toxicity are critical for establishing safe dosing regimens and margins for clinical translation (Table 3).

**Table 3:** Different engineering approaches to improve targeting drug delivery

	Engineering strategy	Description	Advantages	Disadvantages
Overcoming BBB	Ligand Functionalization	Attaching specific ligands (e.g., transferrin, antibodies) to NPs to target receptors on brain ECs [57–61]	Enhanced targeting specificity, improved cellular uptake	High cost, potential off-target effects
	Size Optimization	Engineering NPs within the optimal size range (10–100 nm) for efficient BBB penetration [62]	Facilitates BBB crossing via paracellular and transcellular pathways, enhanced tissue penetration	Larger NPs may face steric hindrance, smaller NPs may be rapidly cleared by renal excretion
	Surface Charge Modification	Altering surface charges to interact with the negatively charged cell membranes [63]	Enhanced cellular uptake, improved stability	Potential cytotoxicity, limited specificity
	Focused Ultrasound (FUS)	Using ultrasound waves to temporarily open the BBB and facilitate NP delivery [64]	Non-invasive, region-specific BBB opening, enhanced permeability for NP-based therapies	Requires optimization for safe BBB disruption, risk of inflammation and tissue damage
Precision Targeting	Magnetic Stimulation	Using magnetic fields to guide magnetic NPs (e.g., SPIONs) to specific brain regions [66–69]	Non-invasive targeting, deep brain penetration, spatial control over NP distribution	Low magnetic susceptibility in brain tissues, potential toxicity of magnetic NPs, limited precision
Controlled Drug Release	Stimuli-Sensitive Coatings	Coatings that respond to pH, enzyme changes, allowing controlled release of drugs in acidic environments (e.g., tumor microenvironment) [73, 74]	Targeted drug release, reduced off-target effects	Limited stability, pH variability *in vivo*
	Light-Responsive NPs	NPs designed to release drugs in response to specific wavelengths of light [75]	Precise spatial and temporal control of drug release, can be non-invasive	Requires external light source, risk of phototoxicity, challenging in deep brain regions
Biocompatibility	PEGylation (Stealth Coating)	Coating NPs with polyethylene glycol (PEG) to increase circulation time and evade immune detection [81, 86]	Prolonged systemic circulation, reduced recognition by immune system, better brain targeting	Possible immunogenicity with repeated dosing, PEG degradation can limit long-term stability
	Biomimetic Coatings	Mimicking natural components of the cell extracellular matrix (e.g., albumin, exosome) [82–85]	Enhanced biocompatibility, improved brain penetration	Complexity in synthesis, potential immunogenicity
	Metal–organic frameworks (MOF)-Based Nanocarriers	Using MOFs for biocompatible, tunable NP designs [77]	Highly customizable, degrades into non-toxic components, stable drug release	Complex synthesis, potential for accumulation in non-target tissues

## Brain-Targeted Drug Delivery Administration Routes

The route of drug administration has emerged not merely as a logistical consideration but as a pivotal determinant of therapeutic efficacy and translational success in neurodegenerative medicine. Current strategies aim to bypass the BBB under two dominating principles: local administration, involving direct invasive delivery into the brain parenchyma or cerebrospinal fluid, and non-invasive systemic administration, where therapeutics enter the bloodstream and cross the BBB via advanced NP platforms. Meanwhile, intranasal delivery has gained substantial traction as a direct, non-invasive route to the brain through the olfactory and trigeminal pathways. This unique pathway effectively bypasses both the BBB and systemic circulation, combining the precision of local delivery with the patient-centric practicality of systemic approaches.

This section delineates the primary NP-based brain delivery routes, examining their underlying anatomical mechanisms, translational feasibility, and evolving role in advancing therapies for neurodegenerative disorders. By weighing the strengths and limitations of each, we show how route selection, integrated with intelligent NP design, can transform clinical outcomes in brain-targeted medicine.

### Local Delivery to the CNS

This method delivers NPs directly into the brain parenchyma or spinal cord via neurosurgical intervention, offering highly targeted delivery to diseased regions. Direct injections have been extensively tested in rodent and primate models of PD and AD [87, 88]. The main techniques are intracerebral, intraventricular, and intrathecal injections.

Intracerebral injection administers drugs directly into the brain parenchyma, bypassing the BBB and localizing therapeutics to diseased regions. This technique is often employed in treating conditions such as glioblastoma, where high local drug concentrations minimize the off-target toxicity of radioactive material [89], and in PD, where targeted delivery to the basal ganglia has shown therapeutic benefit. Yurek *et al*. demonstrated that DNA NPs delivered intracerebrally protected dopaminergic neurons against neurotoxin damage, reaching deep brain structures such as the striatum and substantia nigra [90]. To overcome the physical limitation of poor passive diffusion inherent to this method, recent advancements have paired parenchymal injections with Convection-Enhanced Delivery (CED). CED utilizes a continuous, low-positive pressure gradient via surgically implanted micro-catheters to actively drive NPs through large parenchymal volumes. This mechanical displacement allows macromolecular payloads and complex NPs to achieve homogeneous distribution across expansive, degraded brain regions that standard diffusion cannot reach.

Intraventricular injection delivers drugs into the ventricular system containing CSF. Devices such as the Ommaya reservoir enable repeated dosing and broad distribution via CSF circulation [91]. It is a dome-shaped device inserted underneath the scalp to allow therapeutic agent direct access to CSF under control volume manner.

While the device is an incredibly effective tool for bypassing BBB, it has significant limitations when treating neurodegenerative diseases like AD and PD. These conditions typically require widespread delivery to deep, parenchymal brain structures rather than the fluid spaces. However, moving NPs out of the CSF and into the brain tissue relies almost entirely on passive diffusion and concentration gradient. Thus, the concentration of the therapeutic drops exponentially as it moves further away from the CSF compartment, as well as rapidly clear out from CSF by bulk flow. Unmodified NPs suffer brief retention windows, requiring continuous optimization of surface engineering to promote tissue adherence and high concentrations to achieve effective therapeutic levels.

Intrathecal injection administers agents into CSF via the subarachnoid space around the spinal cord. Though less direct for cortical targeting, it allows drug circulation to the brain and is helpful for conditions such as meningitis or certain cancers affecting the brain surfaces [92]. In modern clinical frameworks, intrathecal delivery has become a critical lifeline for genetic therapies tackling neurodegeneration. It serves as the primary route for antisense oligonucleotides and small interfering RNA nanoplatforms targeting Amyotrophic Lateral Sclerosis (ALS) and HD, capitalizing on direct access to spinal motor neurons and lower motor pathways while avoiding systemic dilution.

While powerful, direct injections are highly invasive, requiring surgery under general anesthesia, drilling into the skull or spine, and highly skilled neurosurgical performance. Risks include bleeding, infection, and localized neuroinflammation that could jeopardize a patient’s life [93]. Some regions remain inaccessible without incurring significant tissue damage, thereby limiting applicability for older ages. Moreover, their invasiveness makes them fundamentally poorly suited for long-term or repeated use, which chronic disorders like PD, AD, HD perpetually demand. Though direct injection offers precise NP delivery for research and select clinical scenarios, its limitations underscore the growing need for non-invasive alternatives that avoid surgical risks while leveraging NP’s ability to cross the BBB.

### Systemic Delivery

#### Oral Delivery

Being a very convenient and non-invasive systemic approach, oral delivery allows for easy administration with NPs taken as pills or capsules in a manner identical to daily medication, thereby significantly improving patient compliance. Schroeder *et al*. reported the successful delivery of drug-loaded Tween 85 NPs to the brain after oral dosing in mice, demonstrating feasibility [94]. However, NPs must pass through the gastrointestinal tract, be absorbed by intestinal walls, and then travel via the bloodstream to the brain. Like IV injection, this route suffers from poor BBB penetration unless NPs are functionalized or combined with BBB-modulating drugs. There are challenges posed to oral delivery in the sense of low bioavailability, resulting from caused by limited dissolution, poor permeability, instability in the gut, and degradation by digestive enzymes [95]. Results from Mittal *et al*. demonstrated brain concentrations of Tween-80 NPs at, approximately 0.3 ng/g (mL) tissue via oral dosing, compared to 1 ng/g (mL) with IV administration (~ 30% efficiency) [96]. To aggressively bypass these severe gastrointestinal and physiological bottlenecks, the paradigm has shifted toward dual-targeting, biomimetic oral nanomedicines. Engineered NPs increasingly leverage receptor-mediated transcytosis pathways across both the intestinal epithelium and the BBB sequentially. For instance, functionalizing NPs with ligands targeting the neonatal Fc receptor (FcRn), vitamin B12 receptors, or transferrin receptors allows the intact carrier to actively "shuttle" across the gut wall into systemic circulation [97]. Encapsulating fragile neuroprotective compounds within lipid-based nano-emulsions or cell-membrane-coated (e.g., exosome-mimetic) shells also protects payloads from enzymatic destruction in the stomach, gradually improving oral delivery from a highly inefficient route into a viable strategy for daily, long-term neurodegenerative maintenance.

#### Intravenous (IV) Injection

This is the most common route of administration for NPs in the treatment of neurodegenerative diseases, as they circulate throughout the body and reach the target tissue via the bloodstream. This approach is more popular than other methods due to its ease of administration. It is simple and low-invasive while yielding a high bioavailability of NP levels to the brain vasculature, like the carotid artery, even with a low dosage [98].

Unlike local injections, IV delivery distributes NPs across multiple brain regions. The evidence from Poduslo and colleagues’ work supported this claim by showing drug accumulation in the cortex, hippocampus, and cerebellum in rats after IV administration [99]. On the other hand, IV delivery suffers from non-specific targeting since NPs circulate systemically, raising risks of off-target effects and toxicity. The BBB also restricts penetration: only ~ 0.11% of therapeutic antibodies reach brain tissue after IV dosing [100]. Achieving the desired therapeutic concentration in the brain requires a higher dose or a more extended treatment period, which risks systemic side effects. In addition, clearance by the RES further limits bioavailability, limiting their effectiveness *in vivo*. While IV injection remains popular for its convenience, optimizing BBB penetration and targeting specificity is crucial for achieving clinical success. To revolutionize the efficiency of IV delivery, contemporary nanomedicine frequently pairs systemic administration with localized physical disruption of the barrier, most notably via Magnetic Resonance-guided Focused Ultrasound combined with intravenous microbubbles [101]. When microbubbles flowing through the brain vasculature are exposed to localized ultrasound waves, they oscillate, safely and transiently opening the tight junctions of the BBB in specific, diseased anatomical regions (e.g., the hippocampus in AD or the substantia nigra in PD). This allows circulating NPs to flood into the target parenchyma at drastically heightened concentrations without altering the systemic dose.

#### Intraperitoneal (IP) Injection

IP injection, which delivers drugs into the peritoneal cavity within the abdominal area, is less common than IV but widely used in animal studies due to ease of administration. The peritoneal cavity is the fluid-filled space surrounding your abdominal and pelvic organs. It allows larger doses and slower, sustained release into circulation, reducing rapid clearance by the liver or kidneys [102]. Typically, when a medication is administered directly into the peritoneal cavity, it is absorbed 25–50% more slowly than when administered intravenously [103, 104]. This is primarily because IP-administered pharmacological agents are absorbed through a large area of the peritoneal cavity then lymphatic system before reaching the bloodstream, which is not as direct as IV injection. The NPs then migrate to the brain by traversing the abdominal vessels and then the internal carotid artery, where they can release their therapeutic payload. NPs delivered via IP injection do successfully reach the brain parenchyma and cross the BBB, but peak brain uptake typically occurs hours after administration rather than immediately [105], suitable for treatment with higher dosage and longer duration.

In contrast to its potential benefits, there are still some obstacles to overcome with IP injection. One challenge is that the NPs need to be able to reach the brain with sufficient dosage after being injected into the peritoneal cavity. Like other systemic circulation-based methods, this approach is not straightforward due to the presence of the BBB, which limits the diffusion of NPs. Another challenge is the pharmacokinetics of NPs. The particles must remain stable in the peritoneal cavity and reach the brain sufficiently. Crucially, while IP injection remains a high-throughput screening tool in rodent models due to its simplicity, its translational viability to human medicine remains heavily restricted. The massive differences in peritoneal fluid volume, surface-area-to-body-mass ratios, and vascular architecture between rodents and humans mean that the slow-release kinetics observed in mice rarely replicate predictably in clinical trials. While preclinical studies demonstrate proof of NPs crossing the BBB [106], the mechanisms underlying this process remain unclear and need further examination to clarify the pharmacokinetics and efficacy of IP injection before translating to clinical application.

### Intranasal Delivery

Intranasal administration facilitates the direct delivery of NPs through the nasal passage into the brain, offering a non-invasive and straightforward method for brain targeting [107]. Intranasal administration effectively delivers various therapeutic agents to the brain, including NPs [108–110]. NPs, typically formulated as sprays or solutions, are absorbed through the nasal mucosa and reach the brain via the olfactory and trigeminal pathways [80, 111]. Consequently, the bioavailability of NPs reaching the brain is higher than that of other administration methods, which is achieved systemically through the vasculature. For example, intranasal delivery of Agomelatine-loaded solid lipid NPs has been shown to bypass the BBB efficiently and increase twofold brain bioavailability compared to IV in the AD model [112]. Similarly, the intranasal administration of rotigotine-loaded chitosan nanoparticles in PD models demonstrated a substantial 11% increase in brain bioavailability, thereby improving the neuroprotective effect in subjects [113].

Furthermore, under this route, NPs can then travel to target specific regions of the brain, such as the hippocampus and cortex [114, 115], which are essential for memory and are often affected in AD. However, drug degradation by nasal enzymes (e.g., cytochrome P450, aminopeptidase) [116, 117] and the nasal cilia's clearance process could shorten drug contact time in nasal cells, resulting in limited absorption [118]. Simultaneously, the non-specific targeting effect that leads to off-targeting also appears in intranasal delivery.

To improve the stability of drugs in the nasal cavity and increase their absorption by the nasal mucosa, a coated delivery carrier is necessary to protect the therapeutic payload from degradation and resist clearance by the nasal cilia. To address these mechanical challenges, modern formulation engineering has introduced smart, thermo-responsive and mucoadhesive *in situ* hydrogels. These polymeric nanocarriers are administered as a free-flowing liquid at room temperature but instantly undergo a phase transition into a viscous gel upon contact with the warm nasal mucosa [119]. This gel state effectively resists mucociliary clearance, providing a prolonged, localized depot over the olfactory epithelium. On the hardware front, translation has been accelerated by precision olfactory-targeting nasal cast spray devices [120]. These advanced devices bypass the respiratory zone entirely and deposit the therapeutic mist exclusively onto the upper olfactory recess, maximizing true nose-to-brain kinetics while minimizing pulmonary off-target exposure.

Overall, each NP delivery route presents its advantages and trade-offs. Oral administration is convenient and compliance-friendly but hampered by gastrointestinal degradation and poor BBB permeability. Direct brain injections allow precise targeting yet are invasive and risky. IV delivery provides broad brain distribution but suffers from off-target toxicity and low BBB penetration. IP injection offers sustained release but remains limited by BBB challenges and uncertain pharmacokinetics. Intranasal delivery bypasses systemic circulation and improves bioavailability, but it faces issues with enzymatic degradation and clearance. Ultimately, the choice of administration route depends on specific therapeutic goals, the nature of the NPs, and the disease being targeted, with intensive research aimed at optimizing each method for improved brain-targeted delivery.

## Predictive Models for Drug Bioavailability and NP Transport

Developing brain-specific NP drug delivery systems hinges on understanding the complex mechanisms governing drug bioavailability and NP transport across the BBB. This is where predictive modeling steps in, like a skilled navigator charting the course through this intricate system. These models provide a sophisticated map by simulating the pharmacokinetics (PK) and pharmacodynamics (PD) of NP-based treatments, thereby helping to optimize delivery strategies. In this section, we will examine the main types of predictive models, their working mechanisms, and their role in advancing brain-targeted therapies.

### PK/PD Model

PK/PD models predict drug concentration over time and its pharmacological effects in the brain by integrating absorption, distribution, metabolism, and excretion (ADME). It operates as an exposure–effect framework, links concentration to functional response, helping estimate whether a drug or nanoparticle formulation is likely to produce a meaningful brain effect.

**Fig. 7:**
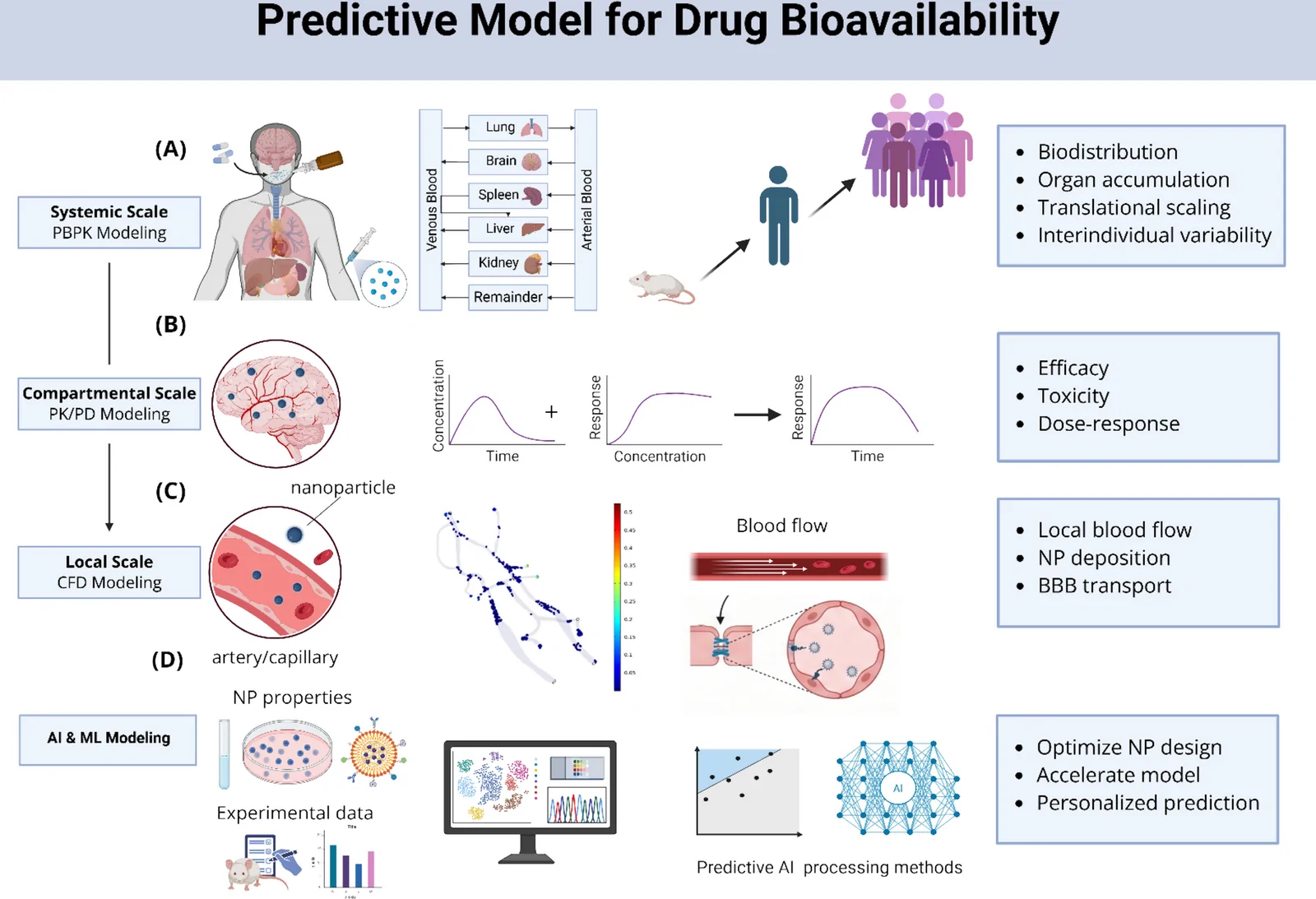
Predictive Models for Brain Drug Bioavailability and NP Transport. (A) PBPK: Systemic drug distribution across major organs. (B) PK/PD: Drug concentration–response relationships over time. (C) CFD: Local NP transport in brain vasculature. (D) AI/ML: Enhances prediction and model refinement across scales.

For NP delivery, modifications to traditional PK/PD models have been made to account for NP’s unique properties, such as size, surface charge, ability to evade immune detection, enhanced permeability and retention (EPR), and receptor-mediated endocytosis at the BBB [121]. Thus, PK/PD models have to explicitly treat the BBB as a transport problem rather than purely a formulation problem. Ranging from one-compartment to more complex multi-compartment systems, the credibility of PK/PD models in NP-based drug delivery is supported by numerous studies that simulate drug release profiles and predict therapeutic outcomes (Fig. 7B). For example, Lindqvist *et al*. employed a two-compartment model to predict the biodistribution and brain penetration of liposome-encapsulated DAMGO that correlated well with experimental data and validated the model's predictive power [122]. Similarly, a study by Arnold *et al*. demonstrated the application of PK/PD modeling in optimizing the dosing regimen of sterically stabilized liposomal doxorubicin for glioblastoma in rats, highlighting the model's capability to enhance therapeutic efficacy while reducing toxicity [123]. The biggest strength of PK/PD is that it can incorporate disease-relevant readouts, which are useful for rank-ordering formulations, optimizing dose, and testing whether improved brain exposure translates into stronger pharmacology. In 2026, work on intranasal cannabidiol-loaded NP, a pharmacokinetic model described rapid brain entry and separated systemic absorption from direct brain entry, showing 47.9% overall bioavailability and 28.5% brain bioavailability *versus* IV administration [124].

However, challenges persist in PK/PD models for capturing NP–BBB complex interactions. As BBB heterogeneity varies across different brain regions, health statuses, ages, genetics, and disease progression, it creates an inter-individual variability that static or uniform models fail to reflect. Most PK/PD studies assume homogeneous BBB permeability, leading to oversimplified compartment predictions when translating from animal models to humans. Despite its critical importance, the complexity of BBB heterogeneity remains underrepresented in the current PK/PD modeling in literature.

For NPs especially, PK/PD is most mature when the formulation has a clear systemic concentration profile and a measurable downstream endpoint, but it is less predictive when transport is dominated by complex biodistribution or BBB interactions. Meanwhile, many also focus on single NP types or disease models, overlooking the broad spectrum of BBB responses in conditions like AD or PD, where permeability is often altered. As a result, these models cannot predict how NPs will behave across diverse pathological states, limiting their applicability to real-world therapeutic development. In addition, the assumption of a uniform NP distribution across brain compartments may not be valid, especially in the presence of non-linear pharmacokinetics. Therefore, while PK/PD models provide a valuable framework for predicting NP’s behavior, their application requires experimental validation and integration with approaches such as physiologically based pharmacokinetic (PBPK) or computational fluid dynamics (CFD) models to capture the full complexity of brain transport and distribution.

### PBPK Models

PBPK models represent an advancement in mechanistic transport and disposition frameworks compared to traditional PK/PD modeling, particularly for therapeutics that must traverse highly restrictive biological boundaries like the BBB. Structurally, PBPK models are compartmental frameworks representing individual organs and tissues connected by blood or lymphatic circulation, simulating ADME processes at the system level through coupled differential equations [125]. By integrating systemic circulation networks with organ-specific kinetics and drug/NP-specific properties, these frameworks are uniquely capable of predicting biodistribution, target exposure, and bioavailability in both the brain and peripheral organs. (Fig. 7A) [126].

While highly effective for small molecules, traditional PBPK models must be significantly adapted to capture the distinct physical reality of a nanocarrier system. Unlike conventional chemical entities, a massive fraction of administered NPs is rapidly opsonized and sequestered by immune cells in the body. To mathematically account for this, modern NP PBPK models bifurcate standard organ compartments into distinct physiological sub-compartments: blood, tissue, and phagocytic cells. Instead of assuming linear uptake, these frameworks utilize non-linear, time-dependent mathematical equations, such as the Hill function [127] or Michaelis-Menten kinetics [128], to simulate the capacity limits and saturation thresholds of macrophages within specific organs.

Beyond structural compartmentalization, recent advancements also emphasize the value of embedding NP-specific critical quality attributes directly into the model equations. For established physical and physiological parameters, such as particle size [126], organ blood flow, and membrane permeability [129], this multi-parametric resolution allows PBPK models to predict complex systemic biodistribution profiles with far greater accuracy than standard PK models. Driven by this success, recent studies have begun attempting to capture the shifting, dynamic chemistry of the nanocarrier *in vivo*. While early frameworks ignored pharmacokinetics concerning surface chemistry modification, protein ligand metabolism, and corona formation due to observational limits, modern iterations are actively trying to integrate empirical terms for phase transitions like particle dissolution, coating degradation, and aggregation [130].

However, extending this to the biological interface remains the field's primary bottleneck. True computational integration of the protein corona is still severely constrained. Because a single nanoparticle in plasma can interact with hundreds of distinct biomolecules, quantitative data governing these precise mechanisms remain scarce, meaning no fully realized computational procedure for corona composition has been successfully standardized in current PBPK workflows.

Meanwhile, a parallel evolution has also occurred in how transport across physical membranes is modeled. Early nanoparticle PBPK studies frequently assumed a "perfusion-limited" framework, treating tissue distribution as instantaneous and governed entirely by blood flow velocity [131, 132]. However, because nanoparticle transport across capillary walls and the dense tight junctions of the BBB is rarely instantaneous, modern approaches increasingly employ permeability-limited or hybrid formulations. Under this paradigm, tissues are split into distinct vascular and extravasation spaces separated by a defined physical membrane barrier [133]. This mathematical bifurcation directly accounts for the reality that a NP’s size, charge, and surface polarity severely hinder its ability to freely traverse cell membranes. Consequently, NP simulations could leverage permeability-limited kinetics to capture precise, localized interactions with the BBB endothelial wall. This ability to map precise mechanical variables allows modern frameworks to achieve another major milestone: simulating why drug exposure differs across routes, species, and disease states, rather than just whether it differs. It is particularly evident in the development of complex multi-route exposure models. In that regard, Bachler *et al*. developed a model for dermal, oral and inhalational exposure to silver ions and Ag NPs [134]. By defining accurate, route-specific uptake rates, these multi-route models provide an unparalleled framework for rigorous inter-route comparisons and safety assessments.

The other ultimate utility of these integrated PBPK frameworks lies in their capacity for translational scaling and personalized medicine. They are particularly valuable for scaling the complex pharmacokinetic effects observed in animal models to humans. Preclinical studies, such as those by Mehta *et al*., have repeatedly demonstrated the effectiveness of PBPK models in translating findings from rats into human clinical contexts, highlighting their role in optimizing drug delivery while minimizing off-target risks [135]. Furthermore, PBPK models are uniquely suited for evaluating interindividual variability to guide personalized treatments. They can programmatically account for patient-specific factors such as age, disease state, and genetic polymorphisms that heavily influence drug transport and efficacy. A key example is the integration of cytochrome P450 genetic variations into PBPK models [136]. Such that these polymorphisms can lead to variable drug responses among individuals, which is critical in conditions like AD and PD. Consequently, the granular simulation allows clinicians to prospectively model and optimize complex dosing regimens for specific, highly vulnerable individuals (e.g., pediatric patients or individuals with multi-organ impairment) before a drug is administered.

Nevertheless, tracking these diverse physiological pathways throughout the whole body requires solving massive systems of coupled equations, a challenge that has driven a simultaneous innovation toward structural simplification. To optimize computational efficiency and reduce this intense parameter bloat, researchers are increasingly adopting "Minimal PBPK" architectures. Instead of mapping the entire anatomy, minimal PBPK models strategically isolate only the plasma and a specific target organ of interest (e.g., isolating the liver for monoclonal antibodies, or the lungs for antituberculosis medications). This streamlined, math-light approach requires significantly less tissue distribution data while maintaining high predictive accuracy for the target site. Recently, Muliaditan *et al*. expanded a minimal PBPK model to characterize TfR-mediated antibody transport across the BBB [137]. By validating the framework against a comprehensive preclinical dataset and human clinical data for trontinemab, the study successfully predicted an optimal non-human primate affinity window from 800 to 3000 nM of dissociation constant for maximized brain delivery.

Reflecting this clinical utility, regulatory agencies are moving from passive acceptance of PBPK data to active enforcement, establishing simulation as a core pillar of Model-Informed Drug Development and Model-Informed Precision Dosing [138]. Major regulatory bodies like the European Medicines Agency (EMA) have formally qualified top-tier simulation platforms (such as Simcyp Simulator) for specific contexts of use. This formal endorsement allows drug sponsors to systematically leverage PBPK simulations to completely waive or replace clinical Drug-Drug Interaction trials, dramatically accelerating drug approval timelines while lowering development costs.

Despite these significant innovations, critical limitations remain. The mathematical accuracy of any PBPK model depends entirely on high-quality input parameters, including blood flow velocities, exact tissue compositions, membrane permeability coefficients, and nanocarrier physical properties like size, surface chemistry, and biodegradation rates [70, 139]. Consequently, NP PBPK models remain highly data-hungry, difficult to validate experimentally, and heavily dependent on physiological assumptions that are not yet fully human-verified. Because high-accuracy parameter data are remarkably difficult to obtain, especially for highly variable, patient-specific contexts [139], PBPK models can still generate predictions that lack precision or fail to adequately reflect real-world physiological conditions. This limitation is particularly stark when extrapolating from animal models to human systems. Much like traditional PK/PD models, current PBPK frameworks still struggle to fully capture the dynamic, structural complexity of the BBB and the shifting, long-term interactions between nanoparticles and diseased brain tissues. Finally, developing, calibrating, and validating these models is highly resource-intensive, demanding extensive paired *in vitro* and *in vivo* data alongside substantial computational power. Recent advancements emphasize that implementing improved, standardized model calibration and validation techniques remains an absolute requirement to enhance the predictive reliability of PBPK models in real-world clinical settings [140].

### CFD Models

Unlike systemic frameworks, CFD models capture the spatial and temporal dynamics of fluid flow and NP transport in localized regions such as the brain vasculature, where fluid motion strongly shapes how much therapeutics actually reach target regions [141–143]. These models provide a detailed analysis of NP transport within the brain's vascular network and interstitial spaces, enabling the simulation of the convective and diffusive behavior of NPs. By adopting continuum approach, CFD integrates fluid dynamics and mass transport equations —e.g., Navier–Stokes for blood flow [142], Darcy’s Law for fluid dynamics in porous media [144], and the species advection-diffusion equation for drug transport [145] to predict NP deposition and distribution across time and space. These models intricately represent the brain's realistic complex geometry, blood flow conditions, interstitial fluid, and BBB properties, making them highly effective for evaluating NP targeting efficiency in distinct brain regions in neurodegenerative disease treatment [70, 146, 147].

A key strength of CFD is that it can resolve real-time physiological conditions, including pulsatile blood flow, shear stresses, and NP–fluid interactions, providing insights into how NPs behave once they are introduced into the bloodstream (Fig. 7C). By doing so, researchers can vividly visualize how NPs are distributed over time and how they penetrate target tissues. This is particularly relevant in neurodegenerative disease, where the therapeutic challenge is often not just getting a drug into the body, but getting it to the correct CNS compartment with adequate residence time. Such understanding is crucial for optimizing personalized patient delivery strategies and ensuring therapeutic agents reach their intended targets.

A representative human in silico cerebrospinal fluid study utilized patient-specific CFD modeling to demonstrate that injection location alters axial tracer distribution to a far greater degree than modest changes in bolus rate or flush volume [148]. Specifically, cisterna magna and intracerebroventricular delivery protocols were shown to increase brain-region area under the curve by approximately 2 logs relative to standard lumbar puncture. This underscores the utility of CFD for protocol optimization, as it can precisely evaluate how individual anatomy and infusion parameters alter regional therapeutic exposure.

Moreover, CFD models are highly customizable and adaptable, enabling researchers to simulate various scenarios by adjusting parameters such as NP size, shape, surface charge, and administration routes [149, 150]. For instance, a recent 2025 meta-analysis of nasal CFD models confirmed that particle size and the related impaction parameter are the most critical determinants of targeting efficiency to the olfactory region, which is essential for bypassing the blood-brain barrier via direct nose-to-brain pathways [151]. This flexibility is particularly beneficial for tailoring NP designs to specific therapeutic goals and optimizing delivery protocols, such as determining the best injection sites and flow rates [152, 153].

Additionally, CFD models provide a non-invasive and cost-effective alternative to early-stage experimental testing, enabling extensive preliminary screening and reducing the need for costly and time-consuming *in vivo* studies.

Recent advancements in CFD have significantly improved its applicability by moving away from generalized models and embracing the natural variability seen in human anatomy and physiology. Leveraging from detailed imaging data of magnetic resonance imaging (MRI) or computed tomography (CT) scans, CFD models can now simulate how NPs navigate through highly specific vasculature, brain tissue, and other regions impacted by neurodegenerative diseases. This allows for tailoring simulations to individual anatomy and physiology, providing more accurate physiological predictions. Studies by Hossain *et al*. [154] and Shamloo *et al*. [155] have demonstrated how such patient-specific CFD models can optimize NP design and predict personalized distribution patterns, ultimately improving the targeting of therapeutic agents. These advantages pave the way for optimizing NP formulations and administration parameters of targeted delivery, thereby enhancing the efficacy of brain-targeted therapies for relevant conditions.

While CFD models have advanced significantly in simulating NPs transport in the brain, these models still grapple with significant hurdles. One of the critical limitations is the lack of accurate and detailed inclusion of the BBB in these simulations. Most existing CFD models miss or oversimplify the BBB, often treating it as a uniform boundary despite well-known regional and pathological-related heterogeneity. For instance, the BBB's permeability may differ in areas like the hippocampus compared to the cortex [156], and even within a single region, permeability can fluctuate based on blood flow and disease progression [157]. As the BBB is often disrupted in diseases like AD or PD, increased permeability is consequential that leads to altered NP transport [158]. This heterogeneity is rarely accounted for in CFD simulations, leading to generalized predictions in drug delivery and may not hold true for all parts of the brain.

Another major challenge is the accurate reconstruction of the brain’s vasculature. The brain’s vascular network is highly intricate, consisting of arteries, veins, capillaries, and the perivascular spaces that serve as crucial pathways for NP movement. Reconstructing the capillary network in CFD models requires high-resolution imaging data from modalities like MRI or CT scans. However, due to their sub-micrometer scale, they often fail to capture fine details of the microvasculature. Imaging artifacts and noise, arising from motion, inadequate contrast, and technological limitations, frequently obscure the visibility of smaller vessels and compromise the model's fidelity. Integrating data across scales from prominent arteries to minute capillaries is fraught with difficulties, requiring sophisticated imaging representation. Moreover, the absence of a universally accepted "gold standard" for brain vasculature reconstructions exacerbates the situation, rendering the validation of emerging methodologies quite problematic. Variability stemming from differing imaging protocols, tissue preparation, and analytical techniques leads to discrepancies in the reconstructed data, ultimately undermining reproducibility and reliability. Since it is geometry and condition-specific, CFD’s prediction is highly sensitive to anatomy, mesh assumptions, boundary conditions, and how well the model captures real physiology. Thus, it should not be used for patient-to-patient prediction, unless paired with high resolution imaging, personalization, or reduced-order models.

​Compounding these challenges, the dynamic of cerebral blood flow is also complex to capture, with existing models often relying on simplified assumptions regarding uniformity and idealized structures.​ Such oversimplifications fail to account for the complex fluid dynamics of varying velocities and pressure gradients in the flow, thereby limiting the application of these models in accurately simulating real-world physiological conditions. This intricate interplay between imaging limitations and computational modeling highlights the critical need for advancement in both areas to enhance the reliability of brain vasculature reconstructions and efficacy in delivery. Additionally, CFD models are also computationally demanding, limiting their real-time clinical use, and require experimental validation to confirm predictions [70, 141]. To overcome these computational bottlenecks, recent workflows have integrated machine learning surrogate architectures, such as random forest and gradient boosting regressors, trained on high-fidelity CFD datasets to enable instantaneous, data-driven predictions of patient-specific regional deposition without the need for prolonged numerical simulation times [159].

Overall, CFD models focus on localized hydrodynamics and regional deposition that appear valuable when the question is route or anatomy-dependent delivery. Their ability to simulate complex transport processes and incorporate patient-specific data makes them instrumental in advancing brain-targeted drug delivery systems. The difference of CFD model keeps it conceptually distinct, while showing how it can be linked with PK/PD and PBPK in a translational pipeline and fully realize their potential in clinical applications.

### Artificial Intelligence (AI)-Driven Models and Machine Learning (ML)

AI/ML for drug bioavailability and nanoparticle transport in neurodegenerative disease is still earlier-stage than PBPK, it is showing great potential in predicting NP pharmacokinetics from physicochemical descriptors and optimizing formulation regardless. Integrating AI and ML algorithms into predictive modeling has accelerated the development of NP delivery systems by identifying optimal combinations of size, shape, surface properties, and drug release profiles. ML algorithms can analyze large datasets generated from experimental and clinical studies to identify complex patterns and predict transport outcomes that are not easily discernible through traditional empirical modeling techniques.

One of the key advantages of AI-driven and ML models is their computing power to process and interpret large volumes of data efficiently. For instance, deep neural networks such as the DeePred-BBB Model have been utilized to predict the BBB permeability of different substances based on their physicochemical properties and molecular fingerprints [160]. By leveraging parallel computing architectures, these deep learning frameworks can screen extensive chemical libraries of over 20,000 small molecules or virtual nanocarriers in a matter of minutes, a high-throughput evaluation scale that would require months of continuous laboratory *in vitro* assays. These advanced algorithms can assess numerous factors simultaneously, such as particle size, shape, surface charge, and chemical composition, to determine their influence on BBB permeability with validated high accuracy (98.07%). Beyond standard deep neural networks, highly specialized architectures are expanding predictive capabilities for central nervous system (CNS) delivery. For example, a recent Information Fusion + Perturbation Theory + Machine Learning (IFPTML) framework, when combined with decision tree algorithms, has achieved up to 96.4% specificity and 79.3% sensitivity in predicting which NP formulations can successfully cross the BBB by merging disparate datasets from drug assays and cytotoxicity studies [161].

Similarly, Farnoud *et al*. has demonstrated the effectiveness of different models, such as Random Forest and Gradient Boosting, in predicting how different NP diameters and flow rates affect the targeting of various regions of nasal epithelium administered via the intranasal route, thereby tuning the design process for brain-targeted drug delivery systems [162]. Furthermore, computational modeling has expanded into predicting the structural stability and self-assembly efficiency of these carriers before they even encounter biological barriers. Techniques like Quantitative Structure-Property Relationship models paired with Support Vector Machines are now used to predict the critical aggregation concentration of amphiphilic peptides, while decision tree variations can rapidly identify how small-molecule drug pairs self-assemble into stable nanocarriers, vastly reducing formulation failures [163].

The predictive power of ML models extends beyond just optimizing NP properties. While these models are not yet fully capable of replicating all the complex dynamics of NP distribution in the brain's microenvironment, they show potential in predicting specific biological interactions related to NP transport within the brain. For instance, deep learning algorithms are being used to model the dynamic transport and distribution of NPs across the brain's environment. These models can predict how NPs accumulate in target tissues, providing valuable insights into the optimal dosing strategies and minimizing off-target effects [164, 165].

Despite their transformative impact, ML and AI-driven models also come with their own set of challenges. They can be sensitive to biases in the training datasets, which are often derived from narrow, restricted chemical libraries or highly specific in silico setups, thus making data pre-processing extremely important. Crucially, models trained and tested purely in silico rarely guarantee comparable performance under complex, real-world biological conditions, as follow-up wet-lab validations or tests across diverse physiological environments remain exceptionally rare in current literature. Additionally, the interpretability of complex AI models, such as deep learning networks, can seem opaque. This lack of transparency can obscure the underlying mechanisms driving predictions, which may hinder the understanding and acceptance of these models in clinical settings. To bridge this gap, the field is beginning to transition away from traditional "black-box" systems toward Explainable AI frameworks and inherently interpretable structures [166]. Researchers are exploring the reduction of architectural redundancies within dense deep learning models to clarify exactly how algorithmic conclusions are reached. Moreover, the next phase of smart drug delivery is poised to leverage physics-informed models, which embed fundamental biophysical laws directly into the machine learning loop, as well as transfer learning and foundation models adapted for materials science, allowing for accurate prediction even when faced with sparse experimental data.

Nonetheless, integrating AI and ML into predictive modeling represents a significant leap forward in developing the NP delivery system for brain-targeted therapies. These technologies can fast-track the entire development process, offering insights that were once impossible to obtain without significant financial and time investments. By systematically integrating laboratory experimental data with high-fidelity in-silico simulations rather than relying on either in isolation, ongoing research can overcome challenges related to data quality, model interpretability, and validation, ultimately enabling the full potential of AI and ML in advancing brain-specific drug delivery systems (Table 4).

**Table 4:** Predictive models for drug bioavailability and NP transport

Model type	Description	Advantages	Challenges
PK/PD Models	Simulate drug ADME and predict drug concentration and effects over time in the brain [121–123]	- Provides time-course predictions for drug effects - Accounts for EPR effects and receptor-mediated endocytosis for NPs	- Oversimplifies BBB heterogeneity - Assumes uniform NP distribution - Limited ability to predict complex NP interactions with BBB
PBPK Models	Use differential equations to model systemic circulation and tissue-specific ADME processes, including the brain and BBB [125, 126, 135, 136]	- Integrates *in vitro* and *in vivo* data - Accounts for inter-individual variability (e.g., age, genetics, disease state) - Helps translate findings from animal models to humans	- Relies on detailed and often hard-to-obtain physiological data - Difficulty in capturing the complexity of BBB and its dynamic interactions
CFD Models	Simulates fluid dynamics and NP transport in brain vasculature, focusing on localized regions [141–143, 149, 150],	- Captures real-time fluid dynamics and shear stresses affecting NP behavior - Patient-specific simulations possible using imaging data	- Often lacks detailed inclusion of BBB - Simplifies vasculature structures - Computationally intensive
AI-Driven and ML Models	Use large datasets to predict NP properties, BBB permeability, and optimize NP designs [160, 162, 164, 165]	- Efficiently processes large, complex datasets - Predicts NP behavior with high accuracy (e.g., BBB permeability) - Optimizes NP size, shape, and surface properties	- Requires high-quality, diverse input data - Limited interpretability of complex models like deep learning - Sensitive to biases in training data

## Clinical Translation of Nanomedicine in Neurodegenerative Diseases

Despite promising preclinical advancements in nanomedicine, the number of approved nano-formulations for central nervous system indications remains disappointingly below projections. This stagnation is primarily due to the notorious translational gap between animal studies and human clinical trials. Promising preclinical results often fail to replicate in patients because of profound interspecies differences in disease progression, metabolic clearance, neuro-vascular architecture, and immune responses, particularly in the context of neurodegenerative diseases. Bridging this gap requires not just incremental refinements to existing protocols, but a foundational paradigm shift toward human-centric preclinical evaluation platforms and advanced, bio-inspired testing methodologies.

### Preclinical Studies and Animal Models for Evaluating Efficacy and Safety

In the realm of neurodegenerative diseases, the translation of nanomedicine from bench to bedside necessitates rigorous, multi-tiered preclinical evaluation. These early-stage studies are crucial for validating the therapeutic index of nanomedicines, utilizing various animal models to assess their efficacy, safety, and pharmacokinetics before progressing to human trials. Animal models, such as transgenic mice and rats, are designed to mimic isolated histopathological features of AD, PD, and HD, providing insights into mechanisms and therapeutic potential.

For example, APP/PS1 transgenic mice, which develop amyloid plaques and cognitive decline, are widely used in AD research. Gold NPs conjugated with anti-Aβ antibodies significantly reduced plaque burden and improved cognition in this model, suggesting promising avenues for AD treatment [167]. Similarly, in PD research, the 6-hydroxydopamine (6-OHDA) rat model mimics the localized dopaminergic neuron loss seen in human PD. Researchers utilize this model to evaluate the neuroprotective effects of nanomedicines designed to mitigate oxidative stress and inflammation. A study by Yeni *et al*. demonstrated that zinc oxide NPs modified with L-DOPA could cross the BBB and prevent neurotoxicity in the 6-OHDA-induced rat, significantly reducing neurodegeneration [168].

Preclinical research requires selecting appropriate animal models that accurately mimic the pathophysiology of neurodegenerative diseases. Evaluation of NP biodistribution, pharmacokinetics, and toxicity profiles in these models provides critical insights into the safety and efficacy of nanomedicine formulations. Meanwhile, behavioral assessments, biochemical analyses, and histological examinations help optimize drug formulations and dosing regimens before advancing to clinical translation.

However, it is crucial to recognize that animal models serve primarily as a schematic biological guide, not as the definitive predictor of human outcomes. Since genetically modified animals only approximate human biology, they naturally fail to capture the complex, age-dependent, multifactorial etiology, and long-term progression of human neurodegenerative diseases. Interspecies variances in microglial activation and systemic immune responses mean that NPs can behave unpredictably in humans, often leading to rapid clearance or unpredicted toxicity that was completely masked in rodent screens.

This translational bottleneck has catalyzed a regulatory shift following the passage of the FDA Modernization Act 2.0, which officially dismantled the absolute mandate for animal testing in preclinical drug development [169]. Consequently, contemporary nanomedicine evaluation increasingly relies on human micro-physiological systems (MPS), such as BBB-on-a-chip models and patient-derived 3D brain organoids. By utilizing human induced pluripotent stem cells (iPSCs) to replicate the shear stress, cellular junctions, and active-transport machinery of the human neurovascular unit, these high-fidelity platforms allow researchers to accurately map human-specific transcytosis and immunotoxicity. Integrating these regulatory-approved, human-centric methodologies prior to *in vivo* scaling provides a more predictive framework, radically filtering out clinical failures at the bench level and streamlining the regulatory approval pipeline for complex CNS nano-formulations. Conversely, because biological MPS operate under a highly controlled microfluidic conditions, translating their raw kinetic data to a full-scale human physiology particularly requires in silico scaling tools. Utilizing multi-compartment PBPK architectures integrated with CFD, researchers can feed parameter inputs derived straight from a BBB-on-a-chip, such as human-specific endothelial permeability coefficients, surface-receptor binding kinetics, and cellular endocytosis rates, into a predictive simulation. These models can simulate macro-scale systemic blood flow, tissue-specific clearance by the RES, and deep parenchymal transport over extended temporal scales. Furthermore, physics-based simulations, such as advection-diffusion and moving mesh mathematics, also allow investigators to model release kinetic and structural variations in the drug vehicle itself (e.g., drug diffusion, matrix degradation rates, delivery path) against the macro-environment of human brain vasculature. By running these mathematical simulations concurrently with microfluidic testing, predictive modeling transforms isolated *in vitro* data points into comprehensive, simulated human biodistribution profiles. This hybrid *in vitro-*in silico approach allows for the high-throughput, virtual optimization of nano-formulations drastically minimizing the reliance on trial-and-error animal scaling and establishing a highly quantified, mathematically sound safety and efficacy profile required by regulatory bodies prior to human clinical entry.

### Regulatory Considerations and Clinical Trial Design for Brain-Specific Drug Delivery

Building on the insights from preclinical studies, translating nanomedicine to the clinic requires comprehensive preclinical data to address the unique challenges of brain-targeted delivery. Regulatory agencies, such as the FDA and EMA, emphasize BBB selectivity, off-target accumulation, and long-term neurotoxicity profiles before approving human trials. Navigating this pathway is uniquely challenging because nanoparticles are classified as combination products, requiring simultaneous validation of both the active drug payload and the synthetic material carrier.

Phase I trials typically focus on safety and dosage, assessing whether nanomedicines can cross the BBB effectively without causing adverse reactions. For example, a Phase I clinical trial tested intranasal insulin for AD (NCT01767909) to explore whether the drug can be safely delivered to the brain through non-invasive means [170]. The candidate must ensure that NPs do not trigger acute anaphylactic or complement-mediated immune responses or accumulate in non-target tissues, prioritizing safety. Nevertheless, challenges in this phase include determining the optimal route of administration and overcoming pathology-driven fluctuations in BBB permeability, which varies drastically among patients and disease states.

As trials progress, Phase II trials aim to establish preliminary efficacy while continuing to assess safety. These trials often focus on a smaller group of patients to evaluate whether the NP drug can achieve the intended therapeutic effects. For instance, a Phase II trial (NCT03993171) investigates whether gold nanocrystal suspension therapy (CNM-Au8, designed to energetically support cellular metabolism and myelination) can enhance drug delivery and support neuroprotection in patients with PD and ALS [171]. However, variability in BBB permeability and disease progression between individuals complicates efficacy assessments, and the long-term impacts of chronic, multi-year NP accumulation in the brain parenchyma are poorly understood. Furthermore, differences in neurodegenerative disease pathology across patients make it challenging to design universally effective treatments, potentially requiring more personalized approaches.

In Phase III trials, larger patient populations are tested to confirm the treatment's efficacy and monitor adverse effects over an extended period. These trials are critical for demonstrating that the drug delivery system consistently provides therapeutic benefits across diverse patient groups. An example is the ongoing trials of Aducanumab (NCT0248454 & NCT02477800), a monoclonal antibody targeting Aβ plaques in AD presented earlier, which leverages advanced drug delivery technologies to optimize its penetration across the BBB [172]. Crucially, modern Phase III evaluation up to 2026 has increasingly adopted adaptive clinical trial designs. These frameworks utilize real-time fluid biomarkers (such as plasma p-tau217 and neurofilament light chain) combined with molecular neuroimaging (PET/MRI) to stratify patient cohorts based on their specific BBB permeability state, allowing investigators to dynamically adjust dosages or delivery schedules mid-trial.

Across all phases, the regulatory approval process must address several key issues: NP biocompatibility, biodistribution, long-term clearance kinetics, degradation byproducts, and the potential for unforeseen side effects such as accelerated neuroinflammation. Current challenges in clinical trial design include developing standardized biomarkers to measure *in vivo* BBB permeability, improving patient selection criteria to account for disease heterogeneity, and ensuring that preclinical data from human-relevant models translate effectively into human outcomes. Addressing these challenges through continued research, bio-inspired engineering, and innovative trial designs is essential for advancing brain-targeted nanomedicines toward widespread clinical use.

## Conclusion & Future Perspectives

Nanomedicine is poised to redefine therapeutic strategies for neurodegenerative diseases, offering unprecedented precision and efficacy in brain-targeted drug delivery. The rapid advancements in NP technology, particularly their ability to cross the formidable BBB, mark a paradigm shift in treating complex neurological disorders. Advances in NP engineering, such as ligand functionalization, surface charge optimization, and stimuli-responsive systems, are addressing challenges of bioavailability and targeting. Crucially, the translation of these complex formulations from benchtop to bedside is increasingly dependent on the strategic integration of predictive computational modeling. Rather than relying on traditional, resource-intensive trial-and-error empirical testing, modern drug delivery pipelines leverage a multi-scale computational framework to systematically optimize delivery efficiency and maximize therapeutic efficacy. At the systemic level, multi-compartment PK/PD and PBPK models allow researchers to mathematically isolate nanoparticle opsonization and simulate permeability-limited transport across the BBB. This drastically enhances efficacy by ensuring that dosing regimens are precisely calibrated to maximize active drug concentrations at the target pathological site while remaining below toxic systemic thresholds. At the local level, high-resolution CFD resolves complex cerebral hydrodynamics and patient-specific microvasculature. By identifying optimal administration routes and flow parameters, CFD helps bypass physical transport barriers to ensure that a significantly higher fraction of the administered dose successfully reaches the deep brain parenchyma. Furthermore, the incorporation of AI and ML algorithms accelerates early-stage development by processing massive molecular datasets, screening virtual nanocarriers for ideal physicochemical descriptors, and accurately predicting BBB penetration within minutes. By linking these distinct computational architectures into an integrated, physics-informed translational pipeline, researchers can quantitatively evaluate how specific critical quality attributes impact long-term bio-distribution and downstream pharmacodynamics. This in silico optimization ultimately minimizes off-target toxicity, ensures adequate therapeutic residence time within diseased central nervous system compartments, and provides a clear mathematical pathway toward model-informed precision medicine. Despite progress, translation to the clinic remains limited by concerns over long-term safety, biocompatibility, and patient variability. The future of brain-specific nanomedicine lies in refining both the design and delivery of NP systems. Biomimetic NPs, modeled after natural carriers such as exosomes or red blood cells, promise enhanced stability, improved circulation, immune evasion, and precise targeting. Similarly, stimuli-responsive “smart” NPs can release drugs in response to pH, enzymes, or light, offering spatial and temporal precision crucial for disorders like AD and PD.

Finally, the path forward must also emphasize the importance of comprehensive preclinical and clinical evaluations. The development of more sophisticated *in vivo* models, including humanized brain organoids and advanced imaging techniques, will be crucial in validating the safety and efficacy of NP-based therapies. Moreover, fostering cross-disciplinary collaboration between bioengineers, clinicians, and computational scientists will be essential to overcome the remaining barriers to clinical translation. With continued innovation and integration across fields, the dream of revolutionizing the treatment of neurodegenerative diseases through NP-based therapies is within reach, offering the promise of symptom management, disease modification, and, ultimately, cures.
